# Effect of midgut proteolytic activity on susceptibility of lepidopteran larvae to *Bacillus thuringiensis* subsp. *Kurstaki*

**DOI:** 10.3389/fphys.2013.00406

**Published:** 2014-01-16

**Authors:** Reza Talaei-Hassanloui, Raziyeh Bakhshaei, Vahid Hosseininaveh, Ayda Khorramnezhad

**Affiliations:** Department of Plant Protection, College of Agriculture and Natural Resources, University of TehranKaraj, Iran

**Keywords:** *Bacillus thuringiensis*, protease activity, toxin activation, resistance, *Plutella xylostella*

## Abstract

*Bacillus thuringiensis* (Bt) is the most effective microbial control agent for controlling numerous species from different insect orders. All subspecies and strains of *B. thuringiensis* can produce a spore and a crystalline parasporal body. This crystal which contains proteinaceous protoxins is dissolved in the alkaline midgut, the resulting molecule is then cleaved and activated by proteolytic enzymes and acts as a toxin. An interesting aspect of this activation process is that variations in midgut pH and protease activity have been shown to account for the spectrum of some Bt proteins activity. Thus, an important factor that could be a determinant of toxin activity is the presence of proteases in the midgut microenvironment of susceptible insects. Reciprocally, any alteration in the midgut protease composition of the host can result in resistance to Bt. Here in this paper, we reviewed this processes in general and presented our assays to reveal whether resistance mechanism to Bt in Diamondback Moth (DbM) larvae could be due to the function of the midgut proteases? We estimated LC_50_ for both probable susceptible and resistant populations in laboratory and greenhouse tests. Then, the midgut protease activities of the *B. thuringiensis* induced-resistant and susceptible populations of the DbM were assayed on Hemoglubin and on N-alpha-benzoyl-DL-arginine-p-nitroanilide (BapNA) for total and tryptic activities, respectively. Six hours after feeding on Bt treated and untreated canola leaves, the midguts of instar larvae of both populations were isolated. Following related protocols, peptides released through the activity of proteinases on Hemoglubin and BApNA were recorded using microplate reader. Control (Blank) was also considered with adding TCA to reaction mix before adding enzymatic extract. Data analysis indicated that there are significant differences for tryptic activity on BApNA and also for total proteolytic activity on Hemoglubin between susceptible and resistant populations fed on Bt treated leaves. But these differences were not significant for larvae fed on healthy canola leaves between these two populations. These results which supported the role of DbM's proteolytic system in development of resistance to Bt, will be discussed in details.

## Introduction

Crops have been beleaguered by insect pests since the beginning of agriculture. Even now, insect herbivory is responsible for nearly 20% of major crop losses worldwide (Ferry et al., 2004, 2006; Mohan et al., 2008). Chemical pesticides have been used to control these herbivores, especially lepidopteran pests, approximately 40% of chemical compounds have been used against caterpillars (Boulter, 1993). As an alternative to chemical control, biological control could be done using predators, parasitoids and entomopathogens (commonly referred as microbial control). Although microbial insecticides have been proposed as substitutes for chemicals but their use is limited since most microbes show a narrow spectrum of activity that enables them to kill only certain insect species (Bravo et al., 2011). But the discovery of the insecticidal activity of *Bacillus thuringiensis* (Bt) launched a new era in pest control, with Bt becoming the leading biopesticide used around the world to combat agricultural pests, predominantly butterflies, moths and beetles and insect vectors of human disease, predominantly aquatic dipteran species. Bt is not a single entity; it is a collection of subspecies and hundreds of isolates that vary widely in their ability to produce a range of toxins and hence have diverse host ranges. Current knowledge on the specificity of Cry toxins is limited to the range of insect species tested in bioassays and the definition of activity. Cry toxicity has been reported for species in six taxonomic orders; Lepidoptera, Coleoptera, Diptera, Hymenoptera, Hemiptera, and Blattaria (van Frankenhuyzen, 2009). It is the most successful insect pathogen used for insect control, presently has almost 2% of the total insecticidal market (Raymond et al., 2010; O'Callaghan et al., 2012).

Bt is a gram-positive, aerobic, facultative anaerobic, endospore-forming bacterium with entomopathogenic properties (Raymond et al., 2009). The production of the characteristic insecticidal (Cry) proteins deposited in crystals in the mother Bt cell has been shown to mainly start from the onset of sporulation. The crystals are composed of millions of Cry (from crystal) or Cyt (cytolytic) toxin molecules. Two main types of Cry proteins are described based on the mass of their protoxin form. The first comprises proteins of 130–140 kDa in mass sharing a highly conserved C terminus containing 15–17 cysteine residues, which is necessary for formation of intermolecular disulfide bonds during crystal formation. Examples of protoxins in this group include Cry1, Cry4A, and Cry4B proteins. The second group of protoxins includes proteins of 70–75 kDa in mass, such as Cry2A, Cry3A, or Cry11A, which do not contain the C-terminal half and are structurally similar to the N-terminal half of the protoxins in the 130-140 kDa toxin group (Jurat-Fuentes and Jackson, 2012).

A number of *cry*-genes have been shown to be transcribed from two overlapping promoters BtI and BtII by RNA polymerases that contain sporulation dependent sigma factors σ E and σ K and a mutation in the consensus region of σ E has been shown to inhibit transcription from BtI and BtII promoters (Sedlak et al., 2000; Perez-Garcia et al., 2010; George and Crickmore, 2012). These crystals are predominantly comprised of one or more proteins (Cry and Cyt toxins), also called δ-endotoxins (Bravo et al., 2007). Bacteria i.e., Bt are unable to penetrate the insect cuticle and can only invade the hemocoel after the gut epithelial barrier is compromised. Thus, the primary route of bacteria entry is the oral cavity during feeding. After having been ingested by susceptible insect larvae and after traversing the peritrophic matrix, *B. thuringiensis* toxins become soluble in the midgut lumen and activated by partial proteolysis. The activated toxins bind to specific receptors, form pores in the apical membranes of the midgut epithelial cells and allow bacteria invasion of the hemocoel, resulting in septicemia (Fortier et al., 2007; Jurat-Fuentes and Jackson, 2012).

*Bacillus thuringiensis* crystalline protoxins are solubilized by the alkaline pH in the midgut of lepidopteran larvae. Solubilization of the protoxin molecules in the crystal renders them available to proteolysis (activation) to yield an active toxin core that is mostly resistant to further proteolysis (Bietlot et al., 1989). Depending on the insect species, protoxins proteolytically activated by midgut proteases (Peyronnet et al., 1997); trypsin-like serin-proteases, elastase-like and chymotrypsin-like proteases. The activated toxin binds to specific receptors on the midgut brush border membrane inducing formation of pores and finally leading to insect death (Schnepf et al., 1998; Budatha et al., 2008). Any disorder in each stage Bt mode of action would be helpful in survival of insect larvae and being resistant of insect population (Tabashnik et al., 1994).

Thus, an important factor that could be a determinant of toxin activity is the presence of proteases in the midgut microenvironment. Numerous potential proteolytic cleavage sites within the activated toxin have been reported (Kirouac et al., 2006).

The continued relevance of Bt toxins in the control of insect and non-insect pests is threatened by the development of resistance by the pests in the field and laboratory reared populations (Tabashnik et al., 1990). There have been reports of insect populations resistant to a particular toxin showing resistance to other toxins to which they have not previously been exposed, a term known as “cross-resistance” (Fabrick and Tabashnik, 2007; Pereira et al., 2008; Gong et al., 2010; Xu et al., 2010). There have been a number of proposed modes of resistance of insect pests to Bt toxins including:

- reduction of binding of toxins to receptors in the midgut of insects,
- reduced solubilisation of protoxin,
- alteration of proteolytic processing of protoxins
- rapid regeneration of the damaged midgut epithelium

(Bruce et al., 2007; George and Crickmore, 2012; Lundgren and Jurat-Fuentes, 2012).

The most studied and experimentally verified mode of resistance is Mode I which is characterized by recessive inheritance, reduced binding by at least more than 500-fold resistance to one Cry1A toxin and little or no cross-resistance to Cry1C. The first gene linked to this Mode I resistance was a cadherin from *Heliothis virescens*. Research results have demonstrated the possibility of cross-resistance development between Cry1Ac and Cry2A by co-occurrence of different mechanisms of resistance in *H. virescens* (Tabashnik et al., 1998; Gahan et al., 2001; Ferre and Van Rie, 2002; Jurat-Fuentes et al., 2003; Lundgren and Jurat-Fuentes, 2012).

Although alteration of the midgut receptors sites even in coleopteran pests (Gao et al., 2011) has received the most attention from researchers, this is not the only mechanism by which insects may evolve resistance to *B. thuringiensis*. Because of the importance of toxin activation during the intoxication process, alterations in the midgut protease composition of the host can result in resistance. For example, resistance to *B. thuringiensis* subsp. *entomocidus* or subsp. *aizawai* in two strains of *Plodia interpunctella* was associated with the loss of a major trypsin-like protease (Oppert et al., 1997).

Changess in protease gene expression in other insect-resistant strains have been reported, but their genetic linkage to resistance has not been established. As activation is a common step in the mode of action of diverse Cry toxins, cross-resistance to other Cry toxins would be expected from alteration of this process. However, not all cases of cross-resistance to diverse toxins correlate with alterations in proteases. For example, a strain of *Spodoptera exigua* selected for resistance with Cry1Ab displayed cross-resistance to toxins not expected to share receptors with Cry1A toxins, such as Cry1D and Cry1Ca, but no protease alterations were detected compared to susceptible larvae. Analysis of *S. exigua* larvae revealed that midgut proliferation was neither increased in resistant insects nor induced by exposure of susceptible larvae to Bt product, suggesting that mechanisms other than midgut proliferation are involved in the response to *B. thuringiensis* by *S. exigua* larvae (Hernandez-Martinez et al., 2008, 2010).

Brush border membrane vesicles from a laboratory selected population of *Ostrinia nubilalis* resistant to Cry1F were found binding the toxin as well as those from a susceptible population and furthermore no differences in activity of luminal gut proteases or proteolytic processing of the toxin were observed (Pereira et al., 2010). This failure to implicate defects in binding or toxin processing in the resistant strain indicates either alternative resistance mechanisms or limitations in the assays used.

Diamondback moth (DbM), *Plutella xylostella* (L.), is a serious and important pest of crucifers in many parts of the world. It was the first crop pest which was reported to be resistant to DDT and now, in many crucifer producing regions, it has shown significant resistance to almost every synthetic insecticide applied in the field. Bt-based products are the most promising alternatives to conventional insecticides because they are highly toxic to certain pests, cause no harm to humans and non-target organisms. The appearance of widespread highly resistance in field was observed in *P. xylostella* population. In *P. xylostella*, the primary resistance mechanism is thought to be reduced binding of Cry1A toxins to the midgut brush border membranes (Tabashnik et al., 1994; Luo et al., 1997; Wright et al., 1997; Sarfraz, 2004).

Laboratory rearing DbM in growth chambers for estimating efficacy of different Bt isolates, we realized that one of our DbM populations finally indicated kind of resistance to a Bt product. Whether this resistance mechanism to Bt in lepidopteran DbM larvae could be due to the function of the midgut proteases? Answering this question, we designed two sets of proteolytic studies to determine total and specific proteolytic activities including both susceptible and resistant populations.

## Materials and methods

DbM larvae and pupae were obtained from cabbage farm around Alborz province by the authors of this article during their previous research. The colony of *P. xylostella* that had been reared on canola plants variety Opera at 25 ± 1°C, 50 ± 5% RH and 16: 8 h L:D showed high susceptibility to a Bt product based on *Bacillus thuringiensis* subsp. *kurstaki*. For investigating the possibility of development of resistance colony, second instar larvae were fed on Bt treated plants in five concentrations of Bt for six generations and the survivals from each generation after maturity produced next generation. Bioassay experiments were carried out separately for susceptible and resistance populations in laboratory and greenhouse. The results indicated that the resistant of *P. xylostella* larvae to *B. thuringiensis* subsp. *kurstaki* 3a3b was faster than excepted.

### Midgut isolation

At first, fourth instar larvae of both populations, susceptible and resistant, were fed on Bt-treated leaves for 6 h (LC_50_ concentrations, Unpublished data), then they were anesthetized and chilled for 15 min and the posterior and interior ends were removed. Guts were excised and midguts were isolated.

### Protease activity assays

This experiment was conducted in two parts, one for the total activity of midgut proteases and at the other part, the trypsin-like enzyme activity in susceptible and resistant population was studied. Six hours post feeding on Bt treated and untreated canola leaves, midguts of 4th instar larvae of both populations were isolated. These midguts were determined as 12 group-assays including whole midgut, midgut wall and midgut contents in each substrate and each population (Bakhshaei et al., 2010).

At the first part of experiment, total proteolytic activity in susceptible and resistant larval gut was measured using hemoglobin (2 mg/ml) as general substrate. In order to prepare enzyme extracts, larval guts were homogenized and the mixture was centrifuged at 13,200 × g, then the supernatant was seperated. One hundred fifty μl from buffer acetate-phosphate-sodium borate, pH 10 was added to microplate containing 50 μl of 2% hemoglobin. Enzyme reaction was started after adding 20 μl enzyme extract and then was incubated for two hours at 30°C. To terminate the reaction, 100 μl of 30% trichloroacetic acid was added to mixture. Then the mixture was chilled at 4°C in order to sediment of non-hydrolyzed substrate. After centrifugation at 16,000 × g for 10 min, the supernatant containing small sequences would be isolated. After adding Folin reagent, the absorbance of releasing peptides because of proteinases action on hemoglobin substrate was measured using with spectrophotometer at 630 nm. Control was conducted by adding trichloroacetic acid to reaction mixture before enzyme extract. This experiment was carried out in three replications.

At the second part of experiment, the activity of trypsin-like enzyme was measured using BApNA as specific substrate in 1 mm final concentration. Enzyme reaction started by adding 20 μl enzyme extract to 5 μl substrate solution in final volume of buffer acetate-phosphate-sodium borate, pH 8.0. The absorbance was monitored at 405 nm with microplate reader. The experiment was performed in three replications. Protease activity values were analyzed by using general linear model (GLM) analysis in three studied sites and binary comparisons were performed using *t*-student test.

## Results and discussion

### Total proteolytic activity

**Figure 1:**
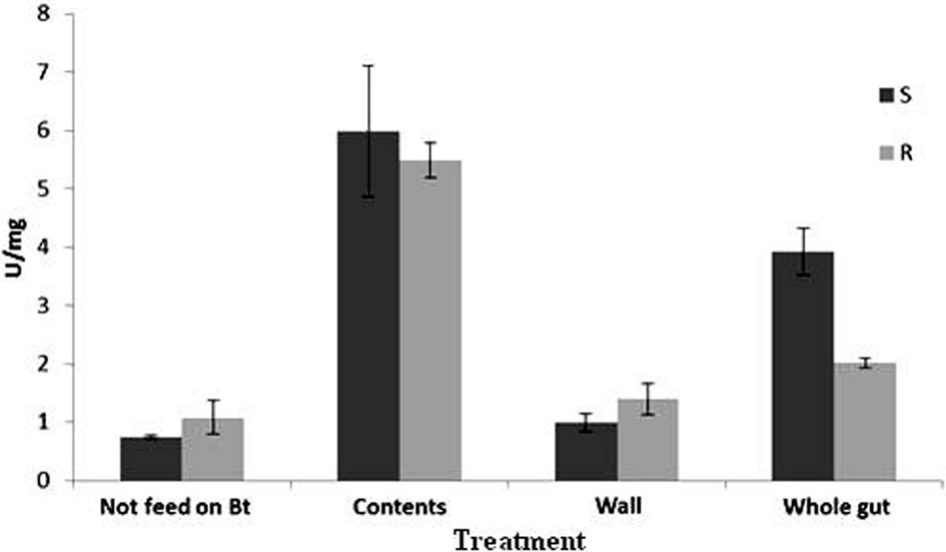
**Mean (±SE) total proteolytic activity for *Plutella xylostella* larvae of resistant (R) and susceptible (S) populations**.

Based on GLM analysis, It was reaveled that there were significant differences (*P* < 0.001) among the treatments for the total protease activity (Figure 1). Mean comparison using *t*-test showed that there was a significant difference between susceptible and resistant populations in proteolytic activity of whole midgut of larvae that were fed on Bt treated leaves for 6 h (*t* = 4.49, *df* = 4, *P* < 0.05), whereas no difference was observed between susceptible and resistant populations in proteolytic activity of whole midgut of larvae that were fed on non-treated leaves (*t* = 1.17, *df* = 4, *P* = 0.31). When proteolytic activity in the gut of susceptible larvae was compared in both cases of feeding, Bt-treated and non-treated leaves, significant difference was recorded between these two cases (*t* = 7.66, *df* = 4, *P* = 0.00) while such comparison for resistant larvae showed no difference (*t* = 1.13, *df* = 4, *P* = 0.32).

### Tryptic activity

**Figure 2:**
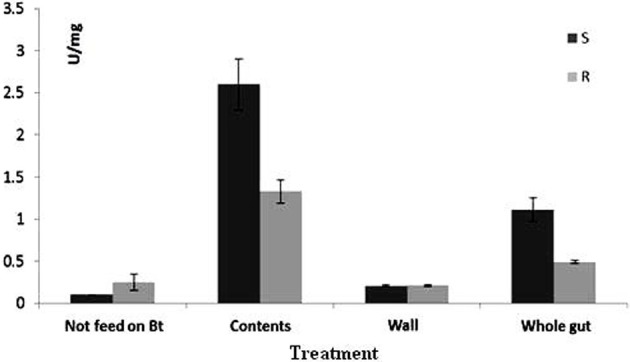
**Mean (±SE) activity of trypsin-like enzyme for *Plutella xylostella* larvae of resistant (R) and susceptible (S) populations**.

GLM results indicated that there were significant differences among the three studied midgut sites for trypsin-like activity (Figure 2) in both susceptible and resistant populations (*P* < 0.001). The interactive effect of these two factors showed also significant difference, which means they didn't act independently. *T*- test comparison showed that there was a significant difference between susceptible and resistant populations in tryptic activity of whole midgut of larvae that had been fed on Bt-treated leaves for 6 h (*t* = 1.7, *df* = 4, *P* = 0.002), while no difference was observed between susceptible and resistant populations in protease activity of whole gut larvae that were fed on non-treated leaves (*t* = 2.61, *df* = 4, *P* = 0.06). When protease activity in the gut of susceptible larvae was compared in both cases of feeding, treated and non-treated leaves, the results revealed significant difference between these two cases (*t* = 12.63, *df* = 4, *P* < 0.01) whereas such comparison for resistant larvae showed no significant difference (*t* = 1.67, *df* = 4, *P* = 0.17).

Based on Tabashnik et al. (1994) results, the main reason of resistance in DbM larvae, was due to reduced or no binding of Bt toxin to receptors on the midgut brush border membrane, while Masson et al. (1995) and Luo et al. (1997) claimed that in some strains of cabbage moth, the rate of toxin receptor binding was the same, but these strains were different in susceptibility to Bt. Therefore there has been no correlation between binding toxin to receptors and toxin insecticidal ability. So the resistant factor was in another point of toxin activation process. Our results revealed that midgut total proteolytic and tryptic activities in susceptible and resistant populations were affected differently. In other words, *P. xylostella* proteolytic system was involved in its resistance to *B. thuringiensis*. Since Bt crystal protein toxicity mechanism has been focused on converting protoxin to active toxin by halfing its molecular weight, it can be interpreted that low activity of tryptic system in resistant population (based on BApNA experiment) reduced drastically this convert. Considering that trypsin-like enzyme activity is predominantly involved in *P. xylostella* larvae for this activation, our results were in conform with Mohan and Gujar (2003), who identified 29.5 kDa trypsin-like protease was the most predominant in activation of protoxins of Cry1Aa and Cry1Ab. But the protoxin and toxin forms of Cry proteins were not different in toxicity toward larvae of *P. xylostella* in their research. Proteolytic activities in *Mamestra brassicae* larval midgut showed that serine proteases were the major activities detected, with chymotrypsin-like and trypsin-like activities being responsible for approximately 62 and 19% of total proteolytic activity toward a non-specific protein substrate (Lightwood et al., 2000; Chougule et al., 2008). But Oppert et al. (2011) indicated that Potato carboxypeptidase reduced the LC_50_ of Cry3Aa for *Rizopertha dominica* two-fold. Their data support the hypothesis that a combination of Cry3Aa protoxin and protease inhibitors, may have applications in control strategies for preventing damage to stored products and grains by coleopteran pests.

Alteration of protease profile in the midgut of Cry1Ac resistant *Helicoverpa armigera* affected the proteolytic processing of Cry1Ac, resulting in the production of 95 and 68 kDa toxins. While an active 65 kDa toxin produced by midgut protease from susceptible population (Rajagopal et al., 2009). This suggests that there is a linkage between improper processing of Bt toxin and development of resistance. Similarly, Sayyed et al. (2005) demonstrated that a field collected resistant population of *P. xylostella* (SERD4) was more sensitive to trypsin-activated Cry1Ab compared to Cry1Ab protoxins. In conclusion, we revealed that our Bt-resistant strain of *P. xylostella* had lower BApNA-hydrolyzing and protoxin-activating abilities than those in susceptible strain. These differences are due to the lack of a major gut trypsin-like proteinase in the resistant strain. Thus, it should be declared that resistance of caterpillars to Bt could be happened easier and faster than that of expected, particularly in some like *P. xylostella*. Although more additional biochemical and molecular studies need to clarify the motives on it and to identify the genes involved in resistance (Sparks et al., 2013), but the proteolytic activity of insect host is an important factor among others. In this regard, frequent applications of Bt formulations which have no adequate spores and include more crystals and also widespread cultivation of Bt plants are serious concerns that must be considered and avoided as possible in management of this possible event.

### Conflict of interest statement

The authors declare that the research was conducted in the absence of any commercial or financial relationships that could be construed as a potential conflict of interest.
